# Design and Synthesis of Novel Triazole-based Peptide Analogues as Anticancer Agents 

**DOI:** 10.22037/ijpr.2019.111722.13320

**Published:** 2019

**Authors:** Maryam Baharloui, Sayed Ahmmad Mirshokraee, Azam Monfared, Mohammad Hassan Houshdar Tehrani

**Affiliations:** a *Department of Chemistry, Faculty of Basic Sciences, Payam Noor University, Tehran, Iran.*; b *Department of Medicinal Chemistry, School of Pharmacy, Shahid Beheshti University of Medical Sciences, Tehran, Iran.*

**Keywords:** Click chemistry, Triazole rings, Peptide analogues, Cancer, MTT assay

## Abstract

Cancer disease is a great concern in the worldwide public health and current treatments do not give satisfactory results, so, developing novel therapeutic agents to combat cancer is highly demanded. Nowadays, anticancer peptides (ACPs) are becoming promising anticancer drug candidates. This is due to several advantages inherited in peptide molecules, such as being usually with small size, high activity, low immunogenicity, good biocompatibility, diversity of sequence, and more modification sites for functionalization. To get benefit of these merits, in this work, we synthesized a new series of triazole- based analogues with peptide scaffold by employing click chemistry and evaluated their anticancer activities against breast, colon cancer cell lines as well as fibroblast cells using MTT assay. Our results suggest that peptide scaffolds containing 1*H*-1, 2, 3-triazole ring group are toxic against colon and breast cancer cells viability, and this effect was more pronounced on MDA-MB-231 cells compared with MCF-7 breast cells. As a conclusion, these designed peptide analogues may be good and safe candidates as future anticancer agents.

## Introduction

Peptide synthesis techniques based on chemical methods have over 100 years of history (1). Peptide-based drugs, due to containing several properties, are becoming an important class of agents used in the pharmaceutical drug market. Peptides are less immunogenic and have potential to penetrate into organs and tissues owing to their smaller size (2). In addition, peptide production affords lower cost and higher benefit for manufactures (2). However, peptides viewed as drugs, generally show low bioavailability and metabolic stability in body and, therefore, are not considered to be very good oral administrated pharmaceutical agents (3). Nonetheless, compared with proteins and antibodies, these disadvantages are not seen formidable and undefeatable obstacles to be overcome and have not inhibited researchers’ interest to focus on the application of modern synthetic techniques in peptide preparation which has dramatically accelerated the development of peptide drugs (4, 5). Furthermore, synthetic peptides, using as therapeutic agents to treat cancer, are gaining momentum in recent years. That is as a result of more information achieved about the cause of anticancer activities of different peptides which is attributed to a variety of mechanisms such as inhibition of angiogenesis, protein-protein interactions, enzymes, proteins, signal transduction pathways, or gene expression (6-13). In view of further application of peptides in the biological system and from the point of gaining benefit from the variety of techniques in peptide synthesis, application of click chemistry has received a great interest among researchers. 

Click chemistry, being rather an attractive approach to the synthesis of chemical scaffolds introduced by Sharpless in 2001, describes a tailoring chemistry to generate new substances quickly and reliably by joining small chemical units together. This is inspired by the fact that nature also generates substances by joining small molecular units (14). Click chemistry has been defined as a reaction that is modular, wide in scope, high yielding, free from offensive byproducts, stereospecific, and simple to perform that requires benign or no solvent (15). Such chemistry has found wide applications not only in synthetic organic chemistry (16), but in dendrimer and polymer chemistry (17), material sciences (18), bioconjugation chemistry (19), and pharmaceutical sciences (20). 

**Figure 1:**
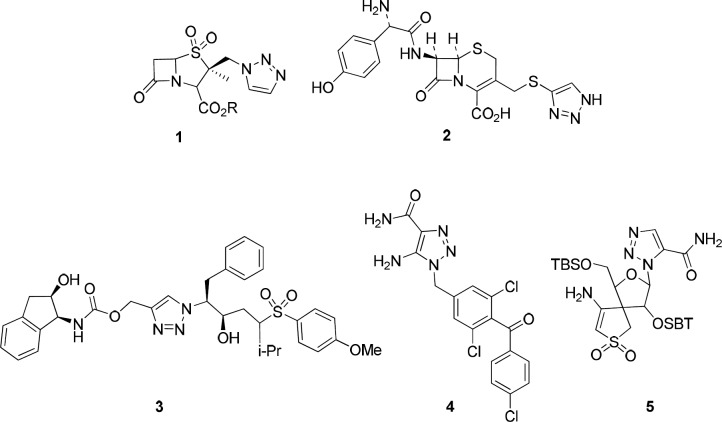
Examples of medicinal 1, 2, 3-triazole ring containing derivatives

From the list of click reactions, there were especial interests in using the Cu(I)-catalyzed variant of the Huisgen 1,3-dipolar cycloaddition of azides and alkynes for the synthesis of 1,4-disubstituted 1,2,3-triazoles, which are important targets for drug discovery (21).1,2,3-Triazoles possess various biological properties including antibacterial, antiallergic, anti-HIV, herbicidal, fungicidal, and anticonvulsant activity (22). Additionally, triazole rings are used as optical brighteners, light stabilizers, fluorescent whiteners, and corrosion retarding agents (23). For example, Tazabactam **1**, Cefatrizine **2**, HIV-1 protease inhibitor **3**, potential anticancer agent **4, **or non-nucleoside reverse transcriptase inhibitor **5** are examples of five-membered ring analogs from 1,2,3-triazole heterocycles (Figure 1) (24-28).

Joining 1, 2, 3-triazole rings and peptide structures possess diverse biological properties including HIV-1 protease inhibition, anticancer potency, and radiolabeling character for tumor diagnosis (3, 29). The present study is aimed to use this approach by synthesizing peptides containing a triazole ring moiety to further explore the anticancer activity of such combination.

## Experimental

*Materials and Methods*

All the chemicals including protected amino acids, Wang resin, and reagents for peptide synthesis were provided from Bachem AG, Switzerland or Santa Cruz Biotechnology Inc; U. S. A. The solvents were purchased from Sigma-Aldrich.

IR spectra of the samples were obtained using a Perkin–Elmer PE 843 IR spectrophotometer, UK. Mass spectra of the samples were recorded on an Agilent 6410 QQQ LCMass spectrometer.

*Synthesis of triazole peptides*

*Preparation of 4-azido benzoic acid*

The azido compound was prepared according to the published method with some modification (30). In brief, 4-aminobenzoic acid (1.64 g, 12.0 mmol) was dissolved in 10 mL water with concentrated hydrochloric acid (6 mL, 12 N). The mixture was stirred at room temperature for 1 h, then cooled to 0-5 °C in ice bath, and to which, an aqueous Na NO_2_ solution (13 mmol, 10 mN) was added dropwise. After 5 min, an aqueous solution of sodium azide (13 mmol, 10 mL) was added to the reaction which was stirred for 5 min. 

The precipitate was isolated by filtration, and extracted with Ethanol (20 mL). The solvent was removed by vacuum to give a light yellow solid (yield 75%). The crude solid was used for the next step of synthesis. IR (KBr): (cm^-1^) 1687, 1700 (C=O carboxylic), 2137(N_3_), 1585, 1613 (aromatic ring). LC-MS (ESI) m/z: 161.8(M-1).

*Preparation 4-(4-phenyl-1H-1, 2, 3-triazol-1-yl) benzoic acid*

**Scheme 1:**
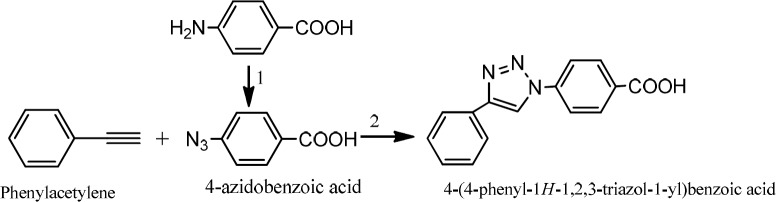
Preparation 4-(4-phenyl-1H-1, 2, 3-triazol-1-yl) benzoic acid. 1) NaNO_2_ /HCl, NaN_3_. 2) CuSO_4_/ sodium ascorbate

The triazole compound was synthesized in accordance with the method previously reported with some changes (Scheme 1) (31).

To a round bottom flask, 4-azidobenzoic acid (1 eq) and phenylacetylene (1 eq) in methanol: water (50:50), sodium ascorbate (50 mg) and CuSO_4_ (13.4 mg) were added in sequence. The reaction was stirred at room temperature overnight. The flask content was poured into water (20 mL) and then extracted with ethyl acetate (3 × 20 mL). The whole organic solvent was washed with saturated NaCl solution (2 × 15 mL) and dried on sodium sulphate powder. After filtration, the solvent was evaporated in vacuum and the crude precipated product was collected (yield 70%). IR (KBr): n (cm ^-1^) 1545, 1594 (aromatic rings), 1420 (N=N). LC-MS (ESI) m/z: 265.9(M+1).

**Figure 2:**
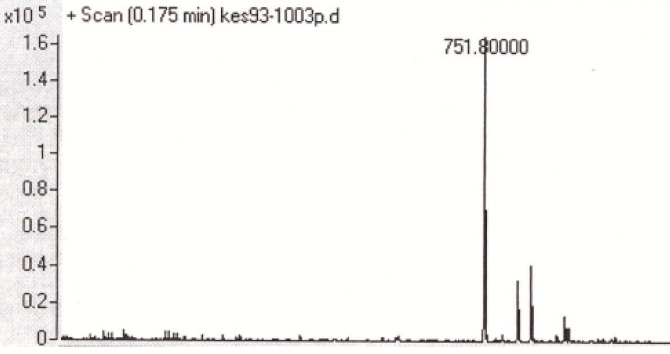
Mass spectrum of the triazole-GLTSK peptide conjugate

**Figure 3:**
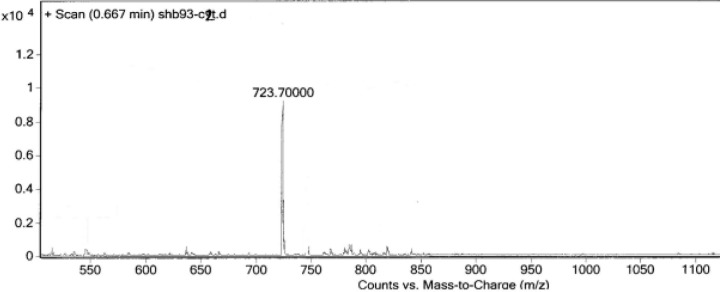
Mass spectrum of the triazole-GEGSGA peptide conjugate

**Scheme 2:**
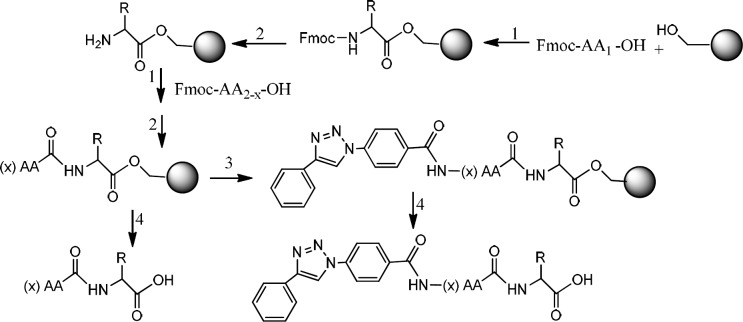
Preparation of triazole peptide conjugate. 1) HOBt, DMAP, DIC, in DMF. 2) Piperazine,in DMF. 3) HOBt, DIC, in DMF. 4) TFA with scavengers

**Table 1:** Anticancer activity of pentapeptides GLTSK (C1), hexapeptide GEGSGA (C2) and their Triazole analogues on Colon cancer cell line HT-29, using MTT assay

	**Colon Cancer cells (HT-29)**	
	**Concentration (µM)**	**Inhibition (%) (Mean ± SD)**
C1 mother	10	93.01 ± 1.01
C1 mother	100	93.06 ± 0.48
C1 mother	1000	92.31 ± 0.16
C1 Triazole	10	92.8 ± 0.16
C1 Triazole	100	92.67 ± 0.45
C1 Triazole	1000	92.36 ± 0.74
C2 mother	10	92.79 ± 0.54
C2 mother	100	93.01 ± 0.57
C2 mother	1000	92.66 ± 0.44
C2 Triazole	10	92.56 ± 0.28
C2 Triazole	100	92.34 ± 0.68
C2 Triazole	1000	91.87 ± 0.39
Ciprofloxacin	10	93.02 ± 0.47
Ciprofloxacin	100	93.01 ± 0.41
Ciprofloxacin	1000	92.56 ± 0.41

**Table 2:** Anticancer activity of pentapeptides GLTSK (C1), hexapeptide GEGSGA (C2), their triazole derivatives on MCF-7, MDA- MB-231 and fibroblast cells (HFF-1), using MTT assay

**Inhibition (%) (Mean ± SD)**			
**Compounds (Concentration 10 µM)**	**MCF-7 cells**	**MDA-MB-231 cells**	**Fibroblast cells**
C1 (mother)	50.11 ± 2.09	78.7 ± 1.60	4.41 ± 2.70
C1Triazole	59.76 ± 10.34	79.9 ± 0.49	3.11 ± 1.19
C2 (mother)	83.39 ± 0.84	79.32 ± 0.23	3.11 ± 1.19
C2Triazole	82.12 ± 0.48	81.22 ± 0.30	5.97 ± 1.62
Ciprofloxacin	76.41 ± 1.08	81.11 ± 0.23	2.33 ± 1.19

*Peptide synthesis on resin*

Two peptides, GLTSK and GEGSGA, previously detected in common bean fractions as inhibitors of human colorectal cancer cells, were synthesized on solid phase method using Wang resin (32). The resin (0.5 g, 1.0-2.5 mmol/g substitution) was swollen in a reactor (fitted at the bottom with a fritted glass filter) by the solvent mixture DMF/ DCM (1: 9, 10 mL) for 1 h and then the solvent was drained off. The first amino acid (2.0 eq), HOBT (2.0 eq) and 4- dimethyl amino pyridine (DMAP) (0.1 eq) in 5 mL DMF were added to the reactor. Diisopropylcarbodiimide (DIC, 1.0 eq) was then added to the reaction vessel and the reactor was shaken for 3 h at room temperature. After 3 h, the mixture was added piperdine/acetic anhydride (2.0 eq: 2.0 eq) and the reaction was stirred for 30 min at room temperature. Following removing solvent by filtration, the resin was washed with DMF (3 × 5 mL), DCM (3 × 5 mL), and methanol (3 × 5 mL). Removing the Fmoc protecting group of the amino acid attached to the resin was performed by treating resin with a solution of piperazine/DMF (10%) for 20 min. The solution was then drained and the resin was washed with DMF (2 × 5 mL). The second amino acid was used with HOBt and DIC (without DMAP) for attaching to the first amino acid bound to the resin. It was followed by washing resin with DMF and DCM. Deprotection was also performed by the *N*-terminal Fmoc removal of the newly formed peptide bound to the resin. Other amino acids were used for bonding to the above peptide linked with resin followed by deprotection, accordingly. 

*Preparation of triazole peptides linked to the resin*

A mixture of the triazole compound, 4-(4-phenyl-1H-1, 2, 3-triazol-1-yl) benzoic acid (2 eq) with HOBt (2 eq) and DIC (2 eq) in 5 mL DMF was prepared and added to a part of peptide linked- resin in the reactor. The reaction was shaken for 3 h at room temperature. Then, the solvent was drained and the resin was washed with DMF (3 × 5 mL) and DCM (3 × 5 mL).

*Cleavage of the peptides from the resin*

Both classes of peptides and triazole conjugated peptides were cleaved from the resin by a solution (10 mL) of trifluoroacetic acid/DCM/anisole /triisopropylsilane (50:45:2.5:2.5) for 2 h. After filtration, the filtrate was added dropwise to an ice-cold diethyl ether. Thus, the precipitated peptides were collected by filtration, washed with cold ether, and kept in a cold and dried condition (Scheme 2).

*Cell toxicity study*

To determine the cytotoxicity of the peptides and their triazole conjugates, three human cancer cell lines were employed; MCF-7 and MDA-MB-231(two breast cancer Cell Lines), and HT-29 (Human Colorectal Adenocarcinoma Cell Line). Human skin fibroblast cell line was also included for comparison. Cell toxicity experiments were carried out in accordance with the previously reported methods with some modification (33, 34). At 37 °C under CO_2_/air (5:95%)_,_ the cells were grown in RPMI1640 medium, enriched with fetal bovine serum (FBS, 10%), penicillin (100 µg/mL), and streptomycin (100 µg/mL). The Cell viability was examined by employing the MTT technique which its principle is on the basis of the transformation of3-(4,5-dimethylthiazol-2-yl)-2, 5-diphenyltetrazolium bromide (MTT) dye to formazan formed as purple crystals by succinate dehydrogenase enzyme of mitochondria in the alive cells. The cells were breeded into 96-well plates at a concentration of 10^4 ^cells/well and incubated for 24 h. The cells were exposed to 10, 100, and 1000 nM concentrations of the peptides for 48 h. MTT (10 μL, 5 mg/mL in PBS) was added to each well at the end of each time analysis, and the microplate was kept at 37 °C for 4 h. 

The medium solution containing MTT was discarded and DMSO (100 μL) was replaced to each well to dissolve the formazan crystals. The plates were then maintained for 20 min at 37 °C. At the end, the optical density of each well was read at 570 nm against the reference wavelength of 630 nm as the background employing a spectrophotometer plate reader (Infinite® M200, TECAN) (35). Ciprofloxacin as a positive cytotoxic control of the peptides was used. Data were shown as the mean of triplicate measuring of the number of living cells.

## Results

Two peptides, GLTSK (C_1_ mother) and GEGSGA (C_2_ mother) were synthesized by solid phase peptide synthesis (SPPS) method using Wang resin with 75% and 80% yield, respectively and their purities were good enough according to the mass spectra results. A part of the each peptide, before cleavage from the resin, was also connected *N*-terminally to 4-(4-phenyl-1H-1, 2, 3-triazol-1-yl) benzoic acid, as shown in Scheme 2 and their mass spectra results gave the appropriate molecular weights, *i.e.*, m/z 751.8(M+1) for triazole-GLTSK and 723.7 (M+1) for triazole-GEGSGA peptide conjugates as shown in Figures 2 and 3, respectively. Anticancer activities of the C_1_ and C_2_ mother peptides were examined on HT-29 colon cancer cell line and the results showed over 90% cell proliferation inhibition using MTT assay (Table 1). 

The C_1 _and C_2_ peptides and their triazole derivatives were also exposed to the breast cancer cell lines MCF-7, MDA-MB-231, as well as the skin fibroblast cells (HFF-1). The results of their anticancer activities, using MTT assay, are given in Table 2.

## Discussion

Click chemistry is a powerful technique for joining various molecular structures to produce chemicals with improved and sometimes with new characteristics to use for diverse applications in biological and pharmaceutical fields (3). Using click chemistry, a triazole scaffold often is produced between two molecular fragments. Molecules containing a triazole ring have many biological activities including anti-inflammatory, antileishmanial, antimicrobial, antitubercular, and anticancer activities (36, 37). Among the biologically active molecules, designing drugs with peptide-based structure containing a triazole ring is an interesting area of research for investigators (3, 29). Such triazole ring containing peptides can also be used in bioconjugation reactions for radiolabelling study in identifying unleashed angiogenesis of tissues *i.e.*, tumors and cancer cells (29). In this study click chemistry was employed to synthesize triazole peptides in order to evaluate their anticancer activities. The 1,2,3 Triazole ring structure as a linker moiety synthesized by click chemistry, as was first reported by Huisgen, needs high temperature applied in the reaction. On the other hand, the reaction produces a mixture of 1,4 and 1,5 disubstituted isomers (38). By discovering copper monovalent (Cu^1^) as a catalyst for such ring formation, the rate of reaction was raised. Moreover, the reaction proceeded regioselectively and produced only 1, 2, 3- triazole with 1, 4-disubstituted isomer (39). In the present study, to construct triazole ring structure, CuSO_4_ along with sodium ascorbate (which give Cu^1^ indigenously insite the reaction) were used (31). Two peptides, GLTSK (C_1_ mother) and GEGSGA (C_2_ mother) were chosen to study their cytotoxic activity, since these peptides, found in four cultivars of common bean, demonstrated antiproliferative activity on some human colon cancer cells (32). The results of MTT assay showed that these peptides were toxic on colon cancer, HT-29 cells, with 10, 100, and 1000 µM concentrations, all with more than 90% activity (Table 1), confirming the previous findings mentioned above. To investigate the cytotoxic activities of these peptides on other tissues, breast cancer cells of MCF-7, MDA-MB-231 were exposed to the low concentrations (10 µM) of the peptides as well as their triazole analogues. Comparing with Ciprofloxacin, as a control drug, cytotoxic activities of these peptides were quite significant. Moreover, triazole derivatives of these peptides preserved and rather raised the cytotoxicity of the peptides. Peptide C_1_ showed toxicity on MCF-7 cells less than MDA-MB-231cells. However, triazole derivative of C_1 _could improve peptide toxicity towards MCF-7 (Table 2). 

Referring to peptide C_2_, although the peptide showed toxic effect on MCF-7 cells higher than C_1 _peptide, C_2 _triazole derivative did not raise the activity of the mother peptide, rather reduced it. Since C_1 _peptide and its triazole derivative carry positive and neutral net charges at physiologic pH (assuming pH 7.2), respectively, and in this regard, C_2_ and its triazole derivative have negative net charges, the aforementioned phenomena can be interpreted that MCF-7 cells preferably select negative rather than neutral ion to allow for entrance. Also, the hydrophobicity of the C_2_ and its triazole conjugate (although with a rather less hydrophobic character) should be high enough to help and force these compounds enter into MCF-7 cells. For more confirmation, it was reported that C_2_ (as being GEGSGA peptide) is more hydrophobic than C_1_ (as being GLTSK peptide) (32). Ciprofloxacin as a control drug demonstrated a lower toxic effect due to its less hydrophobic property as well as being a zwitter ionic molecule. On the other hand, MDA-MB-231cells are promiscuous and accept any kind of positive or negative ionic molecule with moderate hydrophobic character. In this regard, for the more hydrophilic molecule, the chance would be the better for cell entrance (Table 2). It is to be mentioned that although the cytotoxicity of these peptides against the breast cancer cells was generally less than the colon cancer cells, safety profile of these peptides on normal cell lines, HFF-1, could be seen as an advantage. 

## Conclusion

Click chemistry reaction has been largely used in peptide-based drug research. This reaction is very useful for peptide modification hoping to increase the metabolic stability of the peptides. Also, the benefits of this technique are selectivity, efficiency, and mild reaction condition in producing the desired compounds. It is a convenient way to link together two chemicals with different identities, such as peptide fragments with some other functional groups or moieties. In addition, the biological activities of such combinations could be increased. Accordingly, our results indicated that the pentapeptide GLTSK and hexapeptide GEGSGA and their triazole derivatives could be conveniently prepared by solid phase peptide synthesis (spps) method in a short period of time with a high yield. Following examining the cytotoxicity of these synthetic peptides, they exhibited significant anticancer activities against colon and breast cancer cells. Moreover, between two breast cancer cell lines, the peptides and especially their triazole conjugates showed more significant cytotoxic activity on MDA-MB-231 cells rather than on that of MCF-7 cells. Considering the safety profile of these peptides achieved by examining their effects on HFF-1 fibroblast cells, the strategy of synthesizing triazole peptide derivatives may give interesting implications for the construction of structurally diverse heterocyclic molecules which find applications in combinatorial chemistry, diversity oriented synthesis, bioconjugation chemistry, and drug discovery.

## References

[1] Chandrudu S, Simerska P, Toth I (2013). Chemical methods for peptide and protein Production. Molecules.

[2] Vlieghe P, Lisowski V, Martinez J, Khrestchatisky M (2010). Synthetic therapeutic peptides: science and market. Drug Discov. Today.

[3] Li H, Aneja R, Chaiken I (2013). Click chemistry in peptide-based drug design. Molecules.

[4] Merrifield RB (1963). Solid phase peptide synthesis The synthesis of a tetrapeptide. J. Am. Chem. Soc..

[5] Dawson PE, Muir TW, Clark-Lewis I, Kent SB (1994). Synthesis of proteins by native chemical ligation. Science.

[6] Rosca EV, Koskimaki JE, Rivera CG, Pandey NB, Tamiz AP, Popel AS (2011). Anti-angiogenic peptides for cancer therapeutics. Curr. Pharm. Biotechnol..

[7] Karagiannis ED, Popel AS (2008). Novel anti-angiogenic peptides derived from ELR-containing CXC chemokines. J. Cell. Biochem..

[8] Kritzer JA, Stephens OM, Guarracino DA, Reznika SK, Schepartza A (2005). β-Peptides as inhibitors of proteinprotein interactions. Bioorg. Med. Chem.

[9] Mochly-Rosen D, Qvit N (2010). Peptide inhibitors of protein protein interactions: from rational design to the clinic. Chim. Oggi.

[10] Eldar-Finkelman H, Eisenstein M (2009). Peptide inhibitors targeting protein kinases. Curr. Pharm. Des.

[11] Tonelli R, Purgato S, Camerin C, Fronza R, Bolongna F, Alboresi S, Franzoni M, Corradini R, Sforza S, Faccini A, Shohet JM, Marchelli R, Pession A (2005). Antigene peptide nucleic acid specifically inhibits MYCN expression in human neuroblastoma cells leading to cell growth inhibition and apoptosis. Mol. Cancer Ther.

[12] Kakde D, Jain D, Shrivastava V, Kakde R, Patil AT (2011). Cancer therapeutics-opportunities, challenges and advances in drug delivery. J. Appl. Pharm. Sci.

[13] Zheng LH, Wang YJ, Sheng J, Wang F, Zheng Y, Lin XK, Sun M (2011). Antitumor peptides from marine organisms. Mar. Drugs.

[14] Kolb HC, Finn MG, Sharpless KB (2001). Click chemistry: diverse chemical function from a few good reactions. Angew. Chem. Int. Ed.

[15] Kolb HC, Sharpless KB (2003). The growing impact of click chemistry on drug discovery. Drug Discov. Today.

[16] Tilliet M, Lundgren S, Moberg C, Levacher V (2007). Polymer-bound pyridine-bis (oxazoline) Preparation through click chemistry and evaluation in asymmetric catalysis. Adv. Synth. Catal..

[17] Lee JW, Kim JH, Kim BK, Kim JH, Shin WS, Jin SH (2006). Convergent synthesis of PAMAM dendrimers using click chemistry of azide-functionalized PAMAM dendrons. Tetrahedron.

[18] Nandivada H, Jiang X, Lahann J (2007). Click chemistry: versatility and control in the hands of materials scientists. Adv. Mater..

[19] Rozkiewicz DI, Gierlich J, Burley GA, Gutsmiedl K, Carell T, Ravoo BJ, Reinhoudt DN (2007). Transfer printing of DNA by “click” chemistry. Chem. Bio. Chem.

[20] Sharpless K B, Manetsch R (2006). In situ click chemistry: a powerful means for lead discovery. Exp. Opin. Drug Discov.

[21] Nwe K, Brechbiel MW (2009). Growing applications of “click chemistry” for bioconjugation in contemporary biomedical research. Cancer Biother. Radiopharm.

[22] Gil MV, Arevalo MG, Lopez O (2007). Click chemistry: What’s in a name? Triazol synthesis and beyond. Synthesis.

[23] Gouault N, Gupif JF, Sauleau A, David M (2000). ɣ-Methyl-substituted- ɣ-butyrolactones: solid-phase synthesis employing a cyclisation-cleavage strategy. Tetrahedron Lett..

[24] Gnanaprakazam A, Senthi kumar U, Reddy G (2008). Process for the preparation of tazobactam in pure form. United States patent 7417143.

[25] Sultana N, Arayne MS (2007). In-vitro activity of cefadroxil, cephalexin, cefatrizine and cefpirome in presence of essential and trace elements. Pak. J. Pharm. Sci.

[26] Brik A, Muldoon J, Lin YC, Elder JH, Goodsell DS, Olson AJ, Fokin VV, Sharpless KB, Wong CH (2003). Rapid diversity-oriented synthesis in microtiter plates for in situ screening of HIV protease inhibitors. Chem. Bio. Chem.

[27] Soltis MJ, Yeh HJ, Cole KA, Whittaker N, Wersto RP, Kohn EC (1996). Identification and characterization of human metabolites of CAI [5-amino-1-1(4′-chlorobenzoyl-3, 5-dichlorobenzyl)-1, 2, 3-triazole- 4-carboxamide). Drug Metab. Dispos.

[28] Alvarez R, Velazquez S, San-Felix A, Aquaro S, De Clercq E, Perno CFN, Karlsson A, Balzarini J, Camarasa MJ (1994). 1, 2, 3-Triazole-[2,5-Bis-O-(tert-butyldimethylsilyl)-beta-D-ribofuranosyl]-3′-spiro-5′′-(4′′-amino-1′′,2′′-oxathiole 2′′,2′′-dioxide) (TSAO) analogs: synthesis and anti-HIV-1 activity. J. Med. Chem.

[29] Hein CD, Liu XM, Wang D (2008). Click chemistry, a powerful tool for pharmaceutical sciences. Pharm. Res.

[30] Ahmaditaba MA, Houshdar Tehrani MH, Zarghi A, Shahosseini S, Daraei B (2018). Design, synthesis and biological evaluation of novel peptide-like analogues as selective COX-2 inhibitors. Iran. J. Pharm. Res.

[31] Li Z, Yang J, Wang X, Li H, Liu C, Wang N, Huang W, Qian H (2016). Discovery of novel free fatty acid receptor 1 agonists bearing triazole core via click chemistry. Bioorg. Med. Chem.

[32] Luna Vital DA, González de Mejía E, Dia VP, Loarca-Piña G (2014). Peptides in common bean fractions inhibit human colorectal cancer cells. Food Chem.

[33] Rabzia A, Khazaei M, Rashidi Z, Khazaei MR (2017). Synergistic anticancer effect of paclitaxel and noscapine on human prostate cancer cell lines. Iran. J. Pharm. Res.

[34] Mohammadpour F, Ostad SN, Aliebrahimi S, Daman Z (2017). Anti-invasion effects of cannabinoids agonist and antagonist on human breast cancer stem cells. Iran. J. Pharm. Res.

[35] Mosmann T (1983). Rapid colorimetric assay for cellular growth and survival: application to proliferation and cytotoxicity assays. J. Immunol. Methods.

[36] Dheer D, Singh V, Shankar R (2017). Medicinal attributes of 1, 2, 3-triazoles: current developments. Bioorg. Chem..

[37] Haider S, Sarwar Alam M, Hamid H (2014). 1, 2, 3-Triazoles: scaffold with medicinal significance. Inflamm. Cell Signal.

[38] Huisgen R. 1, Padwa A (1984). 3-dipolar cyloaddition chemistry.

[39] Bock VD, Hiemstra H, van Maarseveen JH (2006). CuI -Catalyzed alkyne–azide “Click” cycloadditions from a mechanistic and synthetic perspective. Eur. J. Org. Chem.

